# Comparative Diagnostic Performance of Serum α-Klotho and FGF-23 in Predicting Obstructive Sleep Apnea Severity: A Novel Biomarker Approach

**DOI:** 10.3390/jcm15062316

**Published:** 2026-03-18

**Authors:** Nilgun Erten, Demet Aygun, Aysen Kutan Fenercioglu, Naile Fevziye Misirlioglu, Seyma Dumur, Ulku Dubus Hos, Gonul Simsek, Hafize Uzun

**Affiliations:** 1 Department of Neurology, Istanbul Physical Medicine and Rehabilitation Training and Research Hospital, University of Health Sciences, 34186 Istanbul, Turkey; nderten@hotmail.com; 2 Department of Neurology, Faculty of Medicine, Istanbul Atlas University, 34408 Istanbul, Turkey; 3 Department of Family Medicine, Cerrahpasa Medical Faculty, Istanbul University-Cerrahpasa, 34098 Istanbul, Turkey; aysen.fenercioglu@iuc.edu.tr; 4 Department of Medical Biochemistry, Faculty of Medicine, Istanbul Atlas University, 34408 Istanbul, Turkey; nailemisirlioglu@gmail.com (N.F.M.); seyma.dumur@atlas.edu.tr (S.D.); huzun59@hotmail.com (H.U.); 5 Department of Neurology, İstanbul Başakşehir Çam and Sakura City Hospital, University of Health Sciences, 34480 Istanbul, Turkey; drulku61@gmail.com; 6 Department of Physiology, Faculty of Medicine, Istanbul Atlas University, 34408 Istanbul, Turkey; gdincsimsek@yahoo.com

**Keywords:** obstructive sleep apnea, α-Klotho, FGF-23, inflammation, apnea–hypopnea index, cardiometabolic risk

## Abstract

**Background/Objectives**: Obstructive sleep apnea (OSA) syndrome is characterized by recurrent upper airway obstruction during sleep and is closely associated with systemic inflammation and cardiometabolic risk. α-Klotho and fibroblast growth factor-23 (FGF-23) are emerging biomarkers with potential roles in vascular homeostasis, inflammation, and metabolic regulation. However, their relevance in OSA remains insufficiently elucidated. The aim of this study was to evaluate serum α-Klotho and FGF-23 levels in patients with OSA and to investigate their associations with disease severity. This represents a novel approach that may provide new insights into the pathophysiological mechanisms linking OSA with cardiometabolic risk. **Methods**: A total of 133 participants were included in this study and categorized into three groups according to apnea–hypopnea index: 1—simple snoring (*n* = 44); 2—non-severe OSA (*n* = 44); and 3—severe OSA (*n* = 45). Comparisons between two groups were performed using Student’s *t*-test for normally distributed variables. Comparisons among three or more groups were conducted using one-way ANOVA and the Kruskal–Wallis test. ANCOVA was applied to compare α-Klotho and FGF-23 levels between groups after adjustment for age, BMI, diabetes, hypertension, asthma, COPD, and thyroid disease. The predictive performance of α-Klotho and FGF-23 for severe obstructive sleep apnea was evaluated using ROC curve analysis. **Results**: Serum α-Klotho levels decreased significantly with increasing OSA severity (*p* = 0.001). Serum FGF-23 levels increased significantly across AHI groups (*p* = 0.001). After adjustment for age, BMI, diabetes, hypertension, asthma, thyroid disease, COPD and vitamin D levels, α-Klotho levels were lower in the severe and non-severe OSA group (*p* = 0.001, both) compared to the simple snoring group, whereas FGF-23 levels were higher in the severe and non-severe OSA group (*p* = 0.001; both) compared to the simple snoring group. In predicting the risk of severe OSA compared with non-severe OSA, an α-Klotho cut-off value of 280.3 yielded a sensitivity of 84.44% and specificity of 75%, whereas an FGF-23 cut-off value of 75.5 yielded a sensitivity of 62.2% and specificity of 72.7%. **Conclusions**: Serum α-Klotho levels significantly decrease while FGF-23 levels increase in correlation with OSA severity. α-Klotho exhibited superior predictive performance over FGF-23 in identifying severe OSA, suggesting its potential as a more sensitive biomarker for systemic involvement. These results indicate that the α-Klotho/FGF-23 axis is independently associated with OSA and may play a pivotal role in the pathophysiological mechanisms linking intermittent hypoxia to increased cardiometabolic risk.

## 1. Introduction

Obstructive sleep apnea (OSA) syndrome is a prevalent sleep-related breathing disorder characterized by recurrent upper airway collapse, intermittent hypoxia, and sleep fragmentation. These pathophysiological changes contribute to systemic inflammation, oxidative stress, sympathetic activation, hypertension (HT), metabolic syndrome (MetS), insulin resistance (IR), and cardiovascular disease (CVD) [1]. Despite well-established links between OSAS and cardiometabolic morbidity, the molecular mechanisms underlying this association remain incompletely understood.

Klotho, an anti-aging protein expressed mainly in the kidney and vascular tissues, has emerged as a regulator of oxidative stress, vascular function, and phosphate homeostasis [2]. Circulating soluble α-Klotho levels are decreased in several chronic diseases and have been associated with inflammation and endothelial dysfunction [3,4]. Recent evidence suggests that obstructive sleep apnea (OSA) patients may exhibit reduced α-Klotho concentrations, which negatively correlate with hypoxia severity [5,6]. This supports the hypothesis that α-Klotho could serve as a marker of OSA-related pathophysiology.

Fibroblast growth factor-23 (FGF-23), a hormone secreted by osteocytes, primarily regulates phosphate and vitamin D metabolism. FGF-23 and α-Klotho are key components of the same regulatory axis controlling phosphate and vitamin D homeostasis. α-Klotho acts as an essential co-receptor for FGF-23. In its classic target organs, including the kidney and parathyroid glands, FGF-23 signals through fibroblast growth factor receptor (FGFR)/α-Klotho coreceptor complexes to promote phosphaturia, suppress parathyroid hormone secretion, and reduce active vitamin D levels. In advanced chronic kidney disease (CKD), serum FGF23 rises and α-Klotho declines. Compensatory increases in FGF23 help maintain normal serum phosphate levels despite severely reduced renal function followed by reduced 1,25-(OH)_2_D, increased parathyroid hormone (PTH), and eventually hyperphosphatemia with hypocalcemia [7,8]. OSA may contribute to subclinical renal dysfunction through recurrent intermittent hypoxia, which induces oxidative stress, sympathetic nervous system and renin–angiotensin–aldosterone system activation, leading to glomerular hyperfiltration, endothelial dysfunction, and low-grade renal inflammation before overt declines in glomerular filtration rate become apparent [9].

Beyond mineral balance, FGF-23 has been implicated in cardiovascular remodeling, systemic inflammation, and adverse metabolic outcomes [10]. Given that OSA induces intermittent hypoxia and systemic inflammation, FGF-23 may also be dysregulated in this disorder. Sleep disordered breathing is a common cause of nocturnal hypoxemia that is strongly associated with increased risk of cardiovascular disease. In a community-based analysis of sleep-disordered breathing, Mehta et al. reported that nocturnal hypoxemia was particularly associated with higher circulating C-terminal FGF-23 levels, consistent with experimental evidence indicating that hypoxia can enhance FGF-23 transcription while simultaneously promoting its proteolytic cleavage. These findings support the concept that OSA-related oxygen desaturation may activate the FGF-23 axis even when changes in intact FGF-23 are modest [11]. Mechanistically, intermittent hypoxia in OSA is known to trigger hypoxia-inducible factor (HIF)-mediated stress responses and to increase pro-inflammatory cytokine expression, including interleukin-6 (IL-6) and tumor necrosis factor-α (TNF-α), while inflammation itself has been shown to stimulate FGF-23 production and to correlate with elevated circulating FGF-23 levels in clinical populations [12,13]. In turn, FGF-23 can directly induce inflammatory cytokine production through Klotho-independent pathways, particularly in the liver, suggesting a feed-forward loop in which OSA-induced hypoxemia and inflammation increase FGF-23 levels, thereby further amplifying the inflammatory and cardiovascular risk profile commonly observed in patients with OSAS [10].

Although numerous studies have examined the role of inflammation and oxidative stress in the pathogenesis of OSA, data on specific molecular biomarkers remain limited. Previous research has investigated either α-Klotho or FGF-23 individually in chronic conditions such as chronic kidney disease, cardiovascular disease, and metabolic disorders [1]. To the best of our knowledge, no prior study has comprehensively evaluated serum α-Klotho and FGF-23 concentrations simultaneously in patients with OSA and explored their associations with disease severity. The aim of this study was to evaluate serum α-Klotho and FGF-23 levels in patients with obstructive sleep apnea and to investigate their associations with disease severity. This represents a novel approach that may provide new insights into the pathophysiological mechanisms linking OSA with cardiometabolic risk.

## 2. Materials and Methods

### 2.1. Ethical Approval

The study protocol was developed in accordance with the principles of Good Clinical Practice and the Declaration of Helsinki. Ethical approval was obtained from the Istanbul Atlas University Invasive Scientific Research Ethics Committee (approval no: E-22686390-050.99-73405; date: 5 August 2025). All participants provided written informed consent before participating in this study.

### 2.2. Study Design and Study Population

This study was designed as a prospective case–control study and conducted at the Department of Neurology, Istanbul Atlas University Hospital. A total of 133 participants were included in the study and categorized into three groups according to apnea–hypopnea index: 1—simple snoring (*n* = 44); 2—non-severe OSA (*n* = 44); and 3—severe OSA (*n* = 45).

The diagnosis of OSAS was established based on overnight attended polysomnography (PSG). All participants underwent clinical evaluations, anthropometric measurements, and laboratory assessments.

### 2.3. Inclusion and Exclusion Criteria

Participants aged 18 years or older were eligible for inclusion. Inclusion criteria for the patient group were a confirmed diagnosis of OSA with an apnea–hypopnea index (AHI) ≥ 5 events per hour. Control subjects (simple snoring) were required to have an AHI < 5 events per hour. Only those with a BMI between 18.5 and 39.99 kg/m^2^ were included in the study. According to the WHO classification, individuals were categorized as normal weight with a BMI of 18.5–24.9 kg/m^2^, overweight with a BMI of 25.0–29.9 kg/m^2^, obesity with a BMI of 30.0–39.99 kg/m^2^, and morbid obesity with a BMI ≥ 40.0 corresponding to WHO Obesity Class III [14].

Exclusion criteria included pregnancy, chronic kidney disease, chronic liver disease, malignancy, acute or chronic inflammatory or infectious diseases, other autoimmune disorders, calcium–phosphate metabolism disorders, and the use of medications affecting bone or mineral metabolism. Individuals with a history of thyroid surgery or thyroid malignancy and with a BMI <18.5 and ≥40.0 were also excluded.

### 2.4. OSA Parameter Assessments

Sleep evaluation in patients with OSA was performed using an attended overnight PSG system (Embla Polysomnography System, Model N7000, USA). All recordings were conducted in the sleep laboratory, and each participant underwent full-night PSG monitoring with a minimum duration of 7 h. Standard physiological parameters were continuously recorded, including electroencephalography, electrooculography, electromyography, electrocardiography, airflow, thoracic and abdominal respiratory movements, peripheral oxygen saturation, body position, and snoring. All recorded signals were stored digitally and analyzed using RemLogic software. Automated scoring outputs were subsequently manually reviewed and scored by an experienced sleep technician and independently confirmed by a physician specialized in sleep medicine. OSA was diagnosed with an AHI of ≥5 events per hour. The AHI was calculated as the total number of apnea and hypopnea events divided by the total sleep time in hours. Respiratory events were scored in accordance with the 2012 American Academy of Sleep Medicine (AASM) criteria [15]. Hypopnea was defined as a reduction in airflow of ≥30% from baseline lasting at least 10 s and associated with either a ≥3% decrease in oxygen saturation or an arousal. Apnea was defined as a reduction in airflow of ≥90% from baseline persisting for at least 10 s. The severity of OSA was classified based on the AHI as control (AHI ≤ 5), non-severe (5 ≤ AHI < 30), or severe (AHI ≥ 30). In addition, oxygenation-related parameters derived from PSG reports included total sleep time (TST), percentage of sleep time with oxygen saturation below 90% (T90), mean oxygen saturation (Mean SpO_2_), and minimum oxygen saturation (Min SpO_2_).

### 2.5. Sleepiness Assessment

Excessive daytime sleepiness was assessed using the Epworth Sleepiness Scale (ESS). The ESS is a self-administered questionnaire consisting of eight items designed to evaluate the likelihood of dozing off in common daily situations. Each item is rated on a four-point Likert scale ranging from 0 to 3, yielding a total score between 0 and 24, with higher scores indicating greater daytime sleep propensity. An ESS total score greater than 10 was considered indicative of clinically relevant daytime sleepiness. Based on the total ESS score, participants were stratified into four categories reflecting the severity of daytime sleepiness: individuals with ESS scores of 10 or lower were classified as having no excessive sleepiness, those with scores between 11 and 12 were categorized as having mild sleepiness, scores between 13 and 15 were defined as moderate sleepiness, and scores of 16 or higher were classified as severe daytime sleepiness [16,17,18].

### 2.6. Sample Collection and Biochemical Parameter Assessment

Venous blood samples were collected from all participants after an overnight fast of at least 8 h. Blood samples were centrifuged at 3000 rpm for 10 min to obtain serum, and serum aliquots were stored at −80 °C until analysis. All biochemical analyses were performed in the central biochemistry laboratory of Istanbul Atlas University Hospital under standardized laboratory conditions.

Routine biochemical parameters, including fasting glucose and lipid profiles, were measured using enzymatic colorimetric methods on an automated analyzer (Architect i2000, Abbott Laboratories, Abbott Park, IL, USA). Serum insulin concentrations were determined using the electrochemiluminescence immunoassay (ECLIA) method on a Roche-Hitachi E170 analyzer (Roche/Hitachi MODULAR Analytics Combination Systems, Roche Diagnostics, Indianapolis, IN, USA). Glycated hemoglobin (HbA1c) levels were assessed by high-performance liquid chromatography (HPLC) using the Variant™ Turbo II system (Bio-Rad Laboratories, Hercules, CA, USA).

IR was assessed using the homeostasis model assessment of insulin resistance (HOMA-IR) index, calculated according to the following formula:

HOMA-IR = [Fasting insulin (µU/mL) × Fasting glucose (mg/dL)]/405.

Serum soluble Klotho levels were measured using a commercially available human-soluble Klotho ELISA kit (Catalog No: E-EL-H5451, Elabscience Biotechnology Co., Ltd., Houston, TX, USA). The analytical detection range of the assay was 0.31–20 ng/mL, with a minimum detectable concentration of 0.19 ng/mL, as reported by the manufacturer. Serum fibroblast growth factor-23 (FGF23) concentrations were determined using a human FGF23 ELISA kit (Catalog No: SEA564Hu, Cloud-Clone Corp., Katy, TX, USA). The assay detection range was 15.6–1000 pg/mL, with a sensitivity of <6.1 pg/mL. All ELISA measurements were performed in duplicate according to the manufacturer’s protocols. Intra-assay and inter-assay coefficients of variation were below 10% for both assays. Quality control samples supplied with the kits were included in each analytical run to ensure assay precision and reliability.

### 2.7. Statistical Analysis

Statistical analyses were performed using SPSS software (version 27.0; IBM Corp., Armonk, NY, USA). Quantitative variables are presented as mean ± standard deviation (SD) or median (minimum–maximum), and categorical variables as frequencies and percentages. Data normality was assessed using the Shapiro–Wilk test, skewness–kurtosis values, and Box Plot analysis. Comparisons between two groups were performed using Student′s *t*-test for normally distributed variables. Comparisons among three or more groups were conducted using one-way ANOVA with Bonferroni post hoc analysis for normally distributed data, and the Kruskal–Wallis test with Dunn post hoc analysis for non-normally distributed data. Correlations were analyzed using Pearson or Spearman correlation tests, as appropriate, and linear regression models were used for further analyses. Categorical variables were compared using the chi-square test or the Fisher–Freeman–Halton test. ANCOVA was applied to compare α-Klotho and FGF-23 levels between groups after adjustment for age, BMI, diabetes, hypertension, asthma, COPD, and thyroid disease. The predictive performance of α-Klotho and FGF-23 for severe obstructive sleep apnea was evaluated using ROC curve analysis and the DeLong test. A *p*-value <0.05 was considered statistically significant.

### 2.8. Sample Size/Power Analysis

A post hoc power analysis was performed to evaluate whether the study sample size was sufficient to detect differences in the primary biomarkers across the study groups. The analysis was based on the comparison of serum α-Klotho levels among the three groups defined by obstructive sleep apnea severity (simple snoring, non-severe OSA, and severe OSA) using one-way analysis of variance (ANOVA). Considering the observed group sizes (*n* = 44, 44, and 45) and the reported group means and standard deviations of α-Klotho, the estimated effect size was very large (Cohen’s f ≈ 2.02). With a total sample size of 133 participants and a two-sided significance level of α = 0.05, the achieved statistical power exceeded 99%. A similar calculation performed for FGF-23 levels also demonstrated a very large effect size (Cohen’s f ≈ 1.25) and statistical power greater than 99%. Therefore, the study sample was sufficient to detect clinically meaningful differences in the primary biomarker outcomes among the study groups. Power analysis was performed using GPower software (version 3.1.9.7)

## 3. Results

### 3.1. Participant Characteristics and Polysomnographic Findings

A total of 133 participants were included in this study and categorized into three groups according to AHI: 1—simple snoring (*n* = 44); 2—non-severe OSA (*n* = 44); and 3—severe OSA (*n* = 45). Demographic characteristics were comparable across groups with respect to age, height, weight, body mass index (BMI), and neck circumference (all *p* > 0.05). However, a significant difference in sex distribution was observed (*p* = 0.002), with a higher proportion of females in the non-severe OSA group and prevalence of male participants in the simple snoring and severe OSA group (Table 1).

The frequency of obesity, hypertension, asthma, COPD, and thyroid disease did not differ significantly across AHI groups (*p* > 0.05). The frequency of diabetes mellitus showed a statistically significant difference across AHI groups (*p* = 0.001; *p* < 0.01). The prevalence of diabetes mellitus was higher in both the non-severe and severe OSA groups compared with the simple snoring group (*p* = 0.001 and *p* = 0.001, respectively).

Polysomnographic parameters differed significantly across AHI groups. Mean ODI, T90 (%), ESS scores, and AHI values increased progressively from simple snoring to non-severe and severe OSA groups (*p* = 0.001 for all), while minimum oxygen saturation (Min SpO_2_) decreased significantly with increasing OSA severity (*p* = 0.001). These findings confirm the expected gradation of sleep-disordered breathing severity among the study groups (Table 1).

### 3.2. Laboratory Findings Across AHI Groups

Most metabolic and biochemical parameters, including fasting blood glucose (FBG), insulin, HOMA-IR, renal function tests, liver enzymes, lipid profile, calcium–phosphorus metabolism, hematological indices, and white blood cell counts, did not differ significantly between groups (*p* > 0.05) (Table 2). In contrast, HbA1c levels differed significantly across AHI groups (*p* = 0.001), with both non-severe and severe OSA groups showing higher values than the simple snoring group. CRP values were higher in both the non-severe and severe OSA groups compared with the simple snoring group (*p* = 0.001, all). In addition, CRP levels in the non-severe OSA group were significantly lower than those in the severe OSA group (*p* = 0.001). Vitamin D levels were lower in both the non-severe and severe OSA groups compared with the simple snoring group (*p* = 0.001). Also, vitamin D levels in the non-severe OSA group were significantly higher than those in the severe OSA group (*p* = 0.007). Accordingly, parathyroid hormone (PTH) levels were higher in the severe OSA group compared with the simple snoring group (*p* = 0.005) (Table 2).

### 3.3. α-Klotho and FGF-23 Levels According to OSA Severity

**Figure 1:**
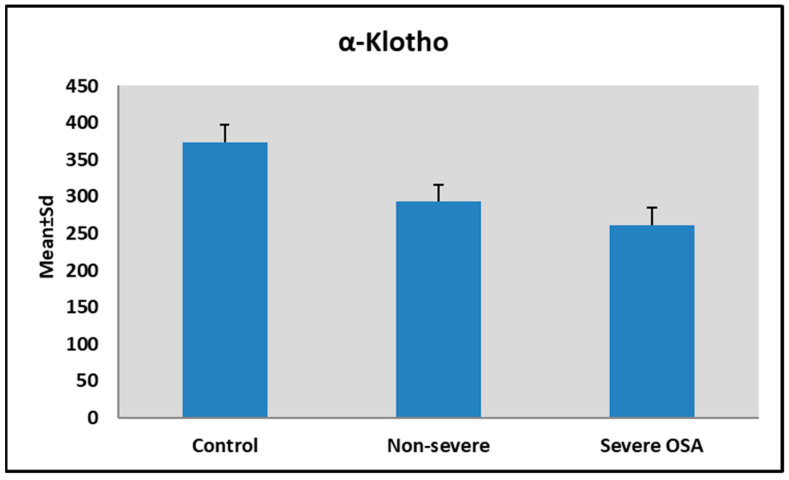
Distribution of α-Klotho levels according to AHI groups.

Serum α-Klotho levels decreased significantly with increasing OSA severity (*p* = 0.001) (Table 2). Both non-severe and severe OSA groups had significantly lower levels than the simple snoring group, and the severe OSA group showed significantly lower levels than the non-severe group (Figure 1).

**Figure 2:**
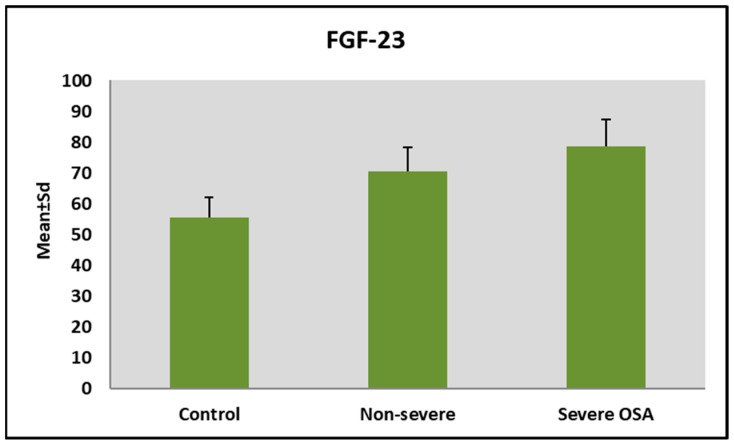
Distribution of FGF-23 levels according to AHI groups.

Conversely, FGF-23 levels increased significantly across AHI groups (*p* = 0.001) (Table 2). Patients with non-severe and severe OSA had significantly higher FGF-23 levels than the simple snoring group, with the highest levels observed in the severe OSA group (Figure 2).

### 3.4. Association of α-Klotho and FGF-23 with Comorbidities

α-Klotho and FGF-23 levels did not differ significantly according to the presence of obesity, hypertension, asthma, COPD, or thyroid disease (*p* > 0.05 for all comparisons), indicating that these biomarkers were not independently influenced by these comorbid conditions (Table 3). However, individuals with diabetes mellitus had significantly lower α-Klotho levels and significantly higher FGF-23 levels compared with those without diabetes (*p* = 0.001; *p* < 0.01).

### 3.5. ANCOVA Analysis

In the constructed model, age, BMI, diabetes mellitus, hypertension, asthma, COPD, and thyroid disease were included as covariates, while the AHI group was entered as the independent variable. After adjustment for age, BMI, diabetes mellitus, hypertension, asthma, COPD, and thyroid disease, the AHI groups remained a strong independent predictor of both α-Klotho and FGF-23 levels (Table 4).

For α-Klotho, the ANCOVA model was statistically significant (F = 54.662, *p* = 0.001; *p* < 0.01) and explained 81.8% of the variance in α-Klotho levels. No statistically significant associations were observed between α-Klotho levels and age, BMI, diabetes, hypertension, asthma, thyroid disease, or vitamin D (*p* > 0.05). However, α-Klotho levels differed significantly according to the presence of COPD (F = 6.677, *p* = 0.011; *p* < 0.05). When the absence of COPD was taken as the reference category, the presence of COPD was associated with lower α-Klotho levels (β = −18.919; *p* < 0.05).

α-Klotho levels differed significantly among the AHI groups (F = 45.505, *p* = 0.001; *p* < 0.01). Compared with the simple snoring group (reference category), α-Klotho levels were on average 108.998 units lower in the severe OSA group (β = −108.998; *p* = 0.001; *p* < 0.01) and 76.443 units lower in the non-severe OSA group (β = −76.443; *p* = 0.001; *p* < 0.01) (Table 4).

The model constructed for FGF-23 levels was statistically significant (F = 22.581, *p* = 0.001; *p* < 0.01) and explained 64.9% of the variance in FGF-23 levels. No statistically significant associations were found between FGF-23 levels and age, BMI, diabetes, hypertension, asthma, COPD, or thyroid disease (*p* > 0.05). However, FGF-23 levels differed significantly among the AHI groups (F = 17.182, *p* = 0.001; *p* < 0.01). Compared with the simple snoring group (reference category), FGF-23 levels were on average 19.998 units higher in the severe OSA group (β = 19.998; *p* = 0.001; *p* < 0.01) and 12.348 units higher in the non-severe OSA group (β = 12.348; *p* = 0.001; *p* < 0.01) (Table 4).

### 3.6. Correlation Analysis

Strong correlations were observed between sleep-disorder breathing severity and biomarker levels. AHI, ODI, T90 (%), and ESS scores were negatively correlated with α-Klotho (r range: −0.746 to −0.798; *p* < 0.01) and positively correlated with FGF-23 (r range: 0.653 to 0.703; *p* < 0.01). Min SpO_2_ showed the opposite pattern, correlating positively with α-Klotho and negatively with FGF-23 (*p* < 0.01 for all). Neck circumference was not correlated with either biomarker (Table 5).

### 3.7. ROC Curve Analysis in Severe OSA Patients

**Figure 3:**
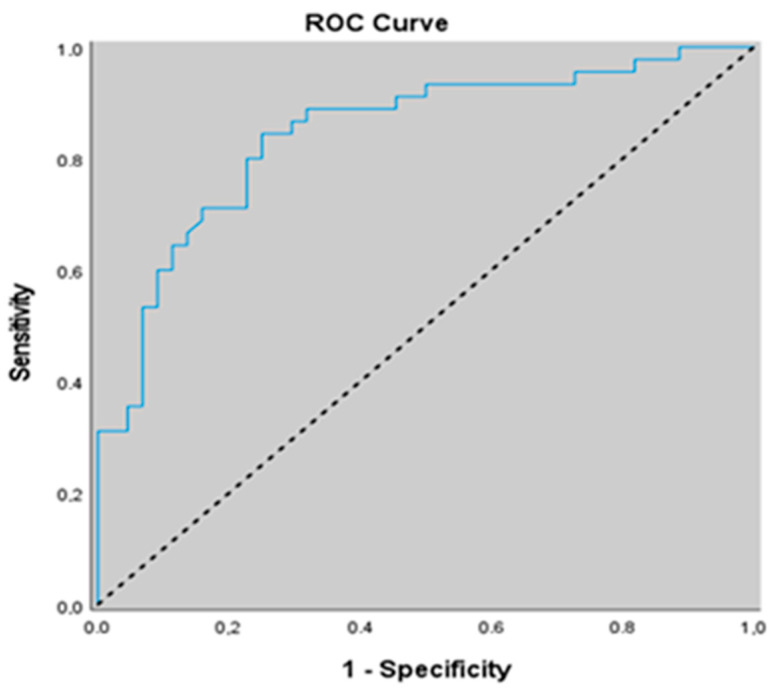
ROC curve of α-Klotho for discriminating severe OSA from non-severe OSA.

Table 6 shows the ROC curve analysis of FGF-23 and α-Klotho in severe OSA patients. In predicting the risk of severe OSA compared with non-severe OSA, an α-Klotho cut-off value of 280.3 yielded a sensitivity of 84.44%, specificity of 75%, positive predictive value of 77.6%, and negative predictive value of 82.5%. The area under the ROC curve was 0.845 with a standard error of 0.042. A statistically significant association was observed between α-Klotho levels ≤280.3 and the risk of severe OSA (*p* = 0.001; *p* < 0.01). Individuals with α-Klotho levels ≤280.3 had a 13.875-fold higher risk of severe OSA positivity (odds ratio: 13.875; 95% CI: 4.981–38.652) (Table 6 and Figure 3).

**Figure 4:**
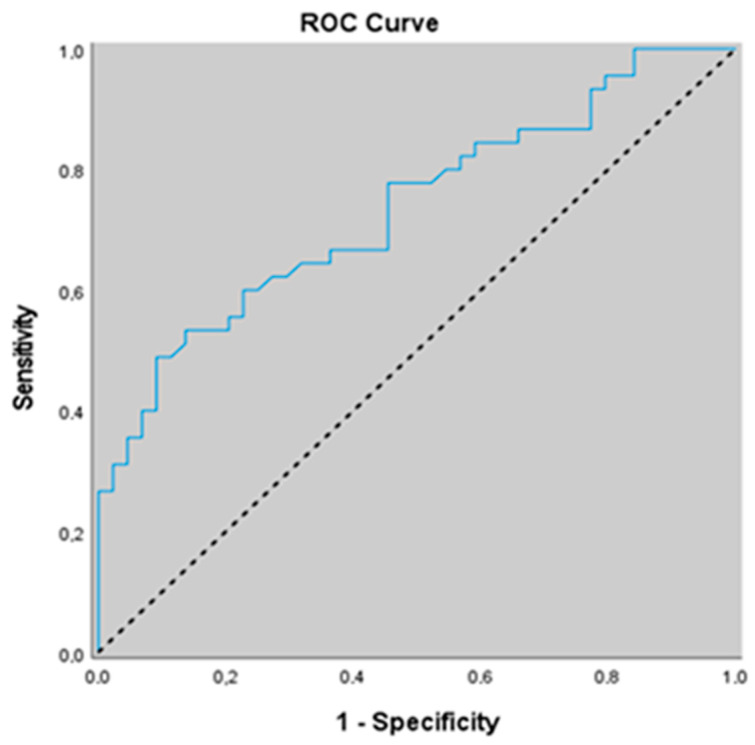
ROC curve of FGF-23 for discriminating against severe OSA from non-severe OSA.

In predicting the risk of severe OSA compared with non-severe OSA, an FGF-23 cut-off value of 75.5 yielded a sensitivity of 62.2%, specificity of 72.7%, positive predictive value of 70.0%, and negative predictive value of 65.3%. The area under the ROC curve was 0.781 with a standard error of 0.042. A statistically significant association was observed between an FGF-23 cut-off value of 78.1 and the risk of severe OSA (*p* = 0.001; *p* < 0.01). Individuals with FGF-23 levels ≥78.1 had a 4.392-fold higher risk of severe OSA positivity (odds ratio: 4.392; 95% CI: 1.792–10.763) (Table 6 and Figure 4).

When the areas under the ROC curves obtained for α-Klotho and FGF-23 in predicting severe OSA versus non-severe OSA were compared using the DeLong test, a statistically significant difference was observed between the two biomarkers (*p* = 0.036; *p* < 0.05), with α-Klotho demonstrating a higher predictive performance than FGF-23 for identifying severe OSA (Figure 3 and Figure 4).

## 4. Discussion

This study identified a clear, severity-dependent disruption of the α-Klotho/FGF-23 axis in OSA. Serum α-Klotho levels progressively declined, whereas FGF-23 levels increased across AHI from simple snoring to non-severe and severe OSA, indicating an opposing biomarker signature that parallels worsening sleep-disordered breathing. Importantly, these differences were not merely driven by baseline anthropometrics, as the groups were comparable in age and BMI, and the AHI category remained an independent and strong predictor of both biomarkers after multivariable adjustment (age, BMI, diabetes, hypertension, asthma, COPD, thyroid disease, and vitamin D). Consistent with a biologically plausible link to intermittent hypoxia, α-Klotho correlated negatively with AHI/ODI/T90 and daytime sleepiness (ESS), while FGF-23 correlated positively with these indices; minimum oxygen saturation showed the inverse pattern. From a clinical discrimination standpoint, α-Klotho outperformed FGF-23 for identifying severe OSA versus non-severe disease (AUC 0.845 vs. 0.735; DeLong *p* = 0.036), and an α-Klotho cut-off ≤280.3 pg/mL provided high sensitivity and specificity. Collectively, these findings support the concept that α-Klotho more than FGF-23 may capture systemic involvement in OSA, offering insight into pathways potentially linking intermittent hypoxia to heightened cardiometabolic risk.

Despite comparable age and anthropometric characteristics across AHI-defined in our cohort, we observed a significant shift in sex distribution, with females over-represented in the non-severe OSA group, whereas males predominated in the simple snoring and severe OSA groups. This pattern is consistent with growing evidence that OSA expression differs by sex, with women often presenting with distinct symptom profiles and potentially different pathophysiologic vulnerability and clinical recognition across the severity spectrum [19]. In addition, diabetes mellitus was markedly more prevalent in both non-severe and severe OSA groups than in the simple snoring group, supporting the well-described bidirectional relationship between OSA and dysglycemia, in which intermittent hypoxia and sleep fragmentation can worsen insulin resistance while diabetes-related mechanisms may also exacerbate sleep-disordered breathing [20,21]. Importantly, our polysomnographic findings demonstrated the expected stepwise deterioration in hypoxemia-related indices (ODI and T90 increasing, minimum SpO_2_ decreasing) with advancing OSA severity; contemporary data increasingly emphasize that metrics capturing hypoxic burden (including T90) may better reflect cardiovascular stress and risk than AHI alone, aligning with studies linking greater nocturnal desaturation exposure to adverse vascular outcomes [22,23]. Finally, the progressive rise in daytime sleepiness (ESS) across AHI groups is directionally consistent with large real-world cohorts showing that excessive daytime sleepiness in OSA correlates not only with event frequency but also with nocturnal oxygenation and desaturation severity [23].

Consistent with the graded polysomnographic deterioration across AHI, our laboratory profile suggested a parallel escalation in metabolic stress and systemic inflammation, despite the absence of broad differences in most routine biochemical parameters. Specifically, HbA1c was significantly higher in both non-severe and severe OSA than in simple snoring, supporting the concept that sleep-disordered breathing is linked to worse long-term glycemic control, likely through intermittent hypoxia-driven sympathetic activation, oxidative stress, and inflammation that promote insulin resistance and impaired glucose homeostasis [24,25,26,27]. In parallel, CRP increased stepwise with OSA severity and was highest in severe OSA, aligning with contemporary systematic review evidence that OSA is associated with elevated circulating CRP and that inflammatory burden tends to track disease severity and hypoxemic exposure [28,29,30,31]. A recent systematic review and meta-analysis by Loh et al. [32] reported that patients with OSA have significantly lower vitamin D levels than healthy controls, and that vitamin D deficiency shows an inverse association with OSA severity. Consistent with these findings, we observed markedly reduced 25(OH) vitamin D levels in OSA, most pronounced in severe disease together with higher PTH in the severe OSA group, a pattern that is biologically plausible given the proposed links between intermittent hypoxia/systemic inflammation and disruption of the vitamin D–PTH axis and bone–mineral metabolism [33,34,35]. In line with this, Siachpazidou et al. [36] also confirmed lower vitamin D levels in patients with OSA; however, they reported that CPAP therapy did not result in a significant increase in serum vitamin D concentrations, suggesting that hypovitaminosis D may reflect broader cardiometabolic and lifestyle-related determinants rather than being solely a direct consequence of sleep-disordered breathing. Notably, the coexistence of higher CRP and lower vitamin D in more severe OSA may represent converging pathways that amplify cardiometabolic risk, reinforcing the need to interpret OSA severity not only by AHI but also within a broader systemic framework [31,33].

Our findings support a severity-dependent dysregulation of the α-Klotho/FGF-23 axis in OSA, characterized by progressively lower α-Klotho and higher FGF-23 concentrations from simple snoring to non-severe and severe disease, and persisting after multivariable adjustment. This pattern is biologically credible because chronic intermittent hypoxia the core pathophysiologic trigger in OSA promotes oxidative stress, endothelial dysfunction, and systemic inflammation, pathways in which Klotho is increasingly regarded as a vasculoprotective and anti-inflammatory modulator. Rostamzadeh et al. [37] synthesized emerging evidence indicating that Klotho-related pathways may be involved in sleep-related cardiovascular phenotypes, supporting the plausibility of Klotho deficiency as a marker of systemic involvement in OSA. Along the same line, Madaeva et al. [38] reported associations between circulating Klotho and sleep/OSA-related parameters, lending clinical support to the notion that reduced Klotho may track disease burden and hypoxemia exposure. In parallel, elevated FGF-23 is increasingly recognized as a stress-responsive hormone with adverse cardiovascular signaling, and Nakano et al. [39] emphasized both direct and indirect cardiac effects of FGF-23, including pro-hypertrophic and pro-fibrotic pathways that may plausibly interact with OSA-related hemodynamic and inflammatory load. Importantly, in our study, α-Klotho and FGF-23 were not materially influenced by obesity, hypertension, asthma, COPD, or thyroid disease, suggesting that the observed biomarker gradients predominantly reflect OSA severity rather than these common comorbidities; however, the significant shift toward lower α-Klotho and higher FGF-23 in diabetes is consistent with the broader metabolic literature. Previous studies have also demonstrated reduced circulating α-Klotho levels in patients with diabetes and insulin resistance, which may be related to chronic inflammation, oxidative stress, and endothelial dysfunction associated with hyperglycemia [40]. Moreover, decreased α-Klotho expression has been linked to accelerated vascular aging and increased cardiometabolic risk in diabetic populations, supporting the hypothesis that diabetes-related metabolic disturbances may further exacerbate the dysregulation of the α-Klotho/FGF-23 axis observed in OSA. In this context, van der Vaart et al. [41] discussed phosphate/FGF-23 perturbations in diabetes and their potential contribution to vascular risk, which may help explain the diabetes-related differences observed in our cohort. Huang et al. [42] reported that FGF-21 is an independent predictor of both prevalent and incident OSA, suggesting that FGF-family hormones may reflect systemic metabolic stress pathways relevant to OSA pathogenesis and progression. This evidence complements our findings on FGF-23 elevation with increasing OSA severity, supporting the concept that dysregulation of endocrine fibroblast growth factors may accompany OSA-related cardiometabolic burden and could have utility for risk stratification. Collectively, these data position the α-Klotho/FGF-23 axis as a promising mechanistic link between intermittent hypoxia and systemic cardiometabolic vulnerability in OSA, meriting validation in longitudinal and CPAP-intervention studies.

The ANCOVA models further indicate that the observed biomarker shifts are robust and largely independent of common clinical confounders. After adjustment for age, BMI, diabetes, hypertension, asthma, COPD, thyroid disease, and vitamin D, the AHI category remained a strong independent determinant of both biomarkers, with an adjusted stepwise reduction in α-Klotho and increase in FGF-23 from simple snoring to non-severe and severe OSA. This supports the interpretation that OSA-related pathophysiology, particularly intermittent hypoxia and its downstream inflammatory/vascular effects, may exert a measurable impact on the α-Klotho/FGF-23 axis beyond background cardiometabolic comorbidity [37]. Notably, COPD emerged as the only covariate independently associated with lower α-Klotho in our adjusted model, which is directionally consistent with population-based evidence linking COPD with reduced circulating Klotho and with the concept of accelerated “inflammaging” in chronic lung disease [43,44]. In contrast, none of the covariates meaningfully explained FGF-23 variability once the AHI group was included, suggesting that the FGF-23 elevation primarily tracks OSA severity in this cohort; this is clinically relevant because higher circulating FGF-23 has been associated with adverse cardiovascular outcomes in meta-analytic evidence, providing a plausible framework by which severe OSA via FGF-23 upregulation could contribute to cardiometabolic risk amplification [45]. From a methodological perspective, our approach aligns with the contemporary OSA biomarker literature that emphasizes covariate-adjusted comparisons to clarify disease-specific effects beyond BMI/sex-related confounding [46].

The correlation and ROC findings provide complementary evidence that α-Klotho and FGF-23 track the hypoxemic and symptomatic burden of OSA, beyond categorical severity definitions. In particular, the strong inverse correlations between α-Klotho and AHI/ODI/T90/ESS, together with the positive correlation with minimum SpO_2_, suggest that progressive nocturnal hypoxemia exposure is closely linked to lower circulating Klotho, consistent with the concept that intermittent hypoxia may downregulate Klotho and contribute to systemic inflammation and vascular vulnerability [47]. Conversely, the positive correlations between FGF-23 and AHI/ODI/T90/ESS and its inverse relationship with minimum SpO_2_ indicate that FGF-23 rises in parallel with worsening oxygen desaturation burden, a clinically relevant observation given meta-analytic evidence linking higher circulating FGF-23 to increased risks of cardiovascular events and mortality in the general population [45]. From a clinical discrimination perspective, ROC analyses further underscore the potential utility of these biomarkers for phenotyping severity: α-Klotho showed higher AUC and a more favorable sensitivity–specificity balance than FGF-23, and the statistically significant DeLong comparison supports a genuine difference in discriminatory performance between the two correlated ROC curves [48]. Taken together, these results align with modern OSA risk frameworks that emphasize the prognostic importance of desaturation-related metrics (e.g., T90/hypoxic burden) and reinforce α-Klotho as a particularly sensitive biomarker candidate for identifying patients with more severe, systemically “loaded” disease [49].

### 4.1. Strengths of the Study

This study offers an original and comprehensive evaluation of the α-Klotho/FGF-23 axis across a clinically relevant OSA spectrum by including simple snoring, non-severe OSA, and severe OSA groups within the same cohort. Beyond AHI-based categorization, the strong associations between these biomarkers and key indices of hypoxemic burden (ODI, T90, minimum SpO_2_), as well as symptom burden (ESS), enhance the biological plausibility of our findings. The use of ANCOVA with multivariable adjustment (age, BMI, diabetes, hypertension, asthma, COPD, thyroid disease, and vitamin D) and the persistence of the AHI group as an independent determinant of both biomarkers further strengthen the robustness of the observed relationships. Finally, the ROC analysis with DeLong comparison demonstrating superior discriminatory performance of α-Klotho over FGF-23 for identifying severe OSA provides clinically meaningful insight and supports potential translational relevance.

### 4.2. Limitations of the Study

The cross-sectional design precludes causal inference; therefore, it cannot be definitively determined whether reduced α-Klotho and increased FGF-23 levels are direct consequences of OSA or reflect parallel systemic processes that co-occur with the condition. Accordingly, the observed associations should be interpreted as correlations rather than evidence of causality. Although a post hoc power analysis indicated that the study sample was sufficient to detect clinically meaningful differences in the primary biomarker outcomes among the study groups, the single-center design and the moderate sample size may limit the generalizability of the findings. Future multicenter studies including larger and more heterogeneous cohorts are needed to validate these findings. In addition, the imbalance in sex distribution across groups may introduce residual confounding despite statistical adjustment. Biomarkers were measured at a single time point, and the absence of longitudinal follow-up or CPAP-intervention data prevents evaluation of treatment responsiveness and prognostic utility. In addition, although renal function was largely within reference ranges, subtle or subclinical renal influences on FGF-23 cannot be entirely excluded.

### 4.3. Clinical Implications

Our results suggest that α-Klotho may serve as a sensitive biomarker for identifying severe OSA and may reflect systemic involvement linked to the hypoxemic burden of sleep-disordered breathing. In clinical practice, α-Klotho (with supportive information from FGF-23) could potentially contribute to risk stratification, particularly in patients with borderline AHI values or disproportionate clinical burden (e.g., prominent sleepiness, marked desaturation, or high cardiometabolic risk). However, before routine implementation, these biomarkers require multicenter validation, assay standardization, and integration into clinically actionable decision frameworks.

### 4.4. Future Directions

Future research should prioritize: (i) external validation in larger, multicenter cohorts; (ii) development of multivariable predictive models incorporating hypoxemia metrics (e.g., T90/ODI or hypoxic burden) alongside AHI; (iii) serial biomarker measurements before and after CPAP to clarify whether α-Klotho and FGF-23 are modifiable with effective therapy; and (iv) prospective studies testing whether these biomarkers predict hard cardiometabolic outcomes (e.g., incident hypertension, subclinical atherosclerosis, or progression of dysglycemia). Integrative analyses combining the α-Klotho/FGF-23 axis with markers of inflammation and endothelial dysfunction (e.g., hs-CRP, IL-6, oxidative stress indices) may further clarify mechanistic pathways linking intermittent hypoxia to increased cardiometabolic risk in OSA.

### 4.5. Conclusions

In this study, circulating α-Klotho and FGF-23 levels displayed a clear severity-dependent pattern across the OSA spectrum, characterized by progressively lower α-Klotho and higher FGF-23 from simple snoring to non-severe and severe OSA. These differences remained significant after adjustment for major clinical confounders, indicating that OSA severity is independently associated with dysregulation of the α-Klotho/FGF-23 axis. Both biomarkers were strongly related to polysomnographic measures of hypoxemic burden and symptom severity, supporting a close link between intermittent hypoxia and systemic biochemical alterations. Importantly, α-Klotho demonstrated superior discriminatory performance compared with FGF-23 for identifying severe OSA, suggesting greater sensitivity as a candidate biomarker for clinically meaningful disease burden. Collectively, our findings highlight the potential of the α-Klotho/FGF-23 axis as a mechanistic pathway connecting OSA-related intermittent hypoxia to heightened cardiometabolic vulnerability and warrant confirmation in larger, longitudinal and CPAP-intervention studies.

## Figures and Tables

**Table 1:** Comparison of participants’ demographics, polysomnography, and sleep characteristics across AHI groups.

		AHI Groups			*p* Value
		Simple Snoring (*n* = 44)	Non-Severe OSA (*n* = 44)	Severe OSA (*n* = 45)	
**Age (Year)**	Mean ± SD	49.00 ± 11.96	51.55 ± 10.13	50.51 ± 11.5	^a^ 0.566
	Median (Min-Max)	48 (30–69)	50.5 (34–68)	53 (30–69)	
**Gender**	**Female (*n* = 60)**	13 (29.5)	29 (65.9)	18 (40)	^b^ ***0.002*** *
	**Male (*n* = 73)**	31 (70.5)	15 (34.1)	27 (60)	
**Height (cm)**	Mean ± SD	166.47 ± 6.19	168.73 ± 8.97	167.81 ± 8.12	^a^ 0.401
	Median (Min-Max)	166.6 (153.6–179.5)	167.5 (149.6–192.6)	168.5 (151.5–184.5)	
**Weight (kg)**	Mean ± SD	78.78 ± 11.86	78.16 ± 14.06	81.67 ± 11.59	^a^ 0.372
	Median (Min-Max)	76.8 (58.9–106.3)	77.9 (53.3–107.7)	82.7 (57.8–114.1)	
**BMI (kg/m^2^)**	Mean ± SD	28.47 ± 4.3	27.53 ± 4.95	29.22 ± 5.06	^a^ 0.253
	Median (Min-Max)	28.7 (20.4–38.5)	27.1 (18.5–38.9)	28.6 (18.6–38.8)	
**Neck circumference (cm)**	Mean ± SD	40.08 ± 4.98	41.62 ± 4.45	39.39 ± 4.55	^a^ 0.073
	Median (Min-Max)	40.3 (32.1–48)	41.3 (32.6–47.9)	39 (32.4–47.7)	
**Diabetes Mellitus (DM)**	**No (*n* = 71)**	40 (90.9)	14 (31.8)	17 (37.8)	^a^ ***0.001*** *
	**Yes (*n* = 62)**	4 (9.1)	30 (68.2)	28 (62.2)	
**Obesity**	**No (*n* = 82)**	30 (68.2)	28 (63.6)	24 (53.3)	^b^ 0.336
	**Yes (*n* = 51)**	14 (31.8)	16 (36.4)	21 (46.7)	
**Hypertension (HT)**	**No (*n* = 91)**	29 (65.9)	29 (65.9)	33 (73.3)	^b^ 0.684
	**Yes (*n* = 42)**	15 (34.1)	15 (34.1)	12 (26.7)	
**Asthma**	**No (*n* = 120)**	5 (11.4)	5 (11.4)	3 (6.7)	
	**Yes (*n* = 13)**	41 (93.2)	40 (90.9)	40 (88.9)	^c^ 0.927
**COPD**	**No (*n* = 121)**	3 (6.8)	4 (9.1)	5 (11.1)	
	**Yes (*n* = 12)**	36 (81.8)	38 (86.4)	40 (88.9)	^b^ 0.628
**Thyroid Disease**	**No (*n* = 114)**	8 (18.2)	6 (13.6)	5 (11.1)	
	**Yes (*n* = 19)**	2.29 ± 1.4	17.34 ± 7.55	53.82 ± 14.22	***N/A***
**AHI**	Mean ± SD	2 (0.2–4.8)	17.7 (5.1–29.7)	55.3 (30.8–78.5)	
	Median (Min-Max)	2.26 ± 2.98	17.83 ± 8.00	52.27 ± 13.9	^a^ ***0.001*** *
**ODI**	Mean ± SD	2.2 (-6.2–8.2)	19.4 (5.2–31.2)	50 (31.7–82.9)	
	Median (Min-Max)	1.82 ± 1.15	12.43 ± 4.24	52.91 ± 44.16	^a^ ***0.001*** *
**T90 (%)**	Mean ± SD	1 (1–5)	11.5 (3–23)	40 (18–288)	
	Median (Min-Max)	95.11 ± 1.90	82.23 ± 4.14	71.71 ± 7.11	^d^ ***0.001*** *
**Min SpO_2_ (%)**	Mean ± SD	95.3 (92.1–98)	82.4 (75.5–89.3)	73 (60.3–84.6)	
	Median (Min-Max)	0.34 ± 0.57	12.5 ± 3.64	16.67 ± 6.02	^a^ ***0.001*** *
**ESS**	Mean ± SD	0 (0–2)	14 (3–18)	17 (0–24)	
	Median (Min-Max)	0 (0–2)	14 (3–18)	18 (0–24)	^d^ ***0.001*** *

^a^ One-way ANOVA test. ^b^ Pearson’s chi-square test. ^c^ Fisher–Freeman–Halton test. ^d^ Kruskal–Wallis test. * *p* < 0.01. SD: standard deviation, Min: minimum, Max: maximum. **AHI**: apnea–hypopnea index, **BMI**: body mass index, **COPD**: Chronic Obstructive Pulmonary Disease, **DM**: diabetes mellitus, **ESS**: Epworth Sleepiness Scale, **HT**: hypertension, **Min SpO_2_**: minimum oxygen saturation, **ODI**: oxygen desaturation index, **OSA**: obstructive sleep apnea, **T90 (%)**: percentage of sleep time with oxygen saturation below 90%.

**Table 2:** Comparison of the laboratory findings across AHI groups.

		AHI Groups			*p* Value
		Simple Snoring (*n* = 44)	Non-Severe OSA (*n* = 44)	Severe OSA (*n* = 45)	
**FBG (mg/dL)**	Mean ± SD	130.71 ± 26.16	129.63 ± 32.1	130.44 ± 28.32	0.984
	Median (Min-Max)	130.7 (80.6–179.9)	131.2 (80.5–178.7)	131.5 (83.7–177.1)	
**HbA1c (%)**	Mean ± SD	5.63 ± 0.59	7.15 ± 1.2	7.04 ± 1.29	***0.001*** **
	Median (Min-Max)	5.6 (4.7–7.5)	7.5 (4.9–8.8)	6.6 (4.9–9)	
**Insulin (µIU/mL)**	Mean ± SD	14.58 ± 6.2	12.95 ± 6.38	14.3 ± 6.7	0.447
	Median (Min-Max)	14.8 (3.7–24.9)	13.6 (3.2–24.7)	13.8 (3.7–24.7)	
**HOMA-IR**	Mean ± SD	4.6 ± 2.03	4.25 ± 2.60	4.65 ± 2.51	0.7
	Median (Min-Max)	4.3 (1.1–9.2)	3.9 (0.9–10.7)	4.3 (1–10.6)	
**Urea (mg/dL)**	Mean ± Sd	30.98 ± 11.39	29.35 ± 12.67	29.19 ± 11.55	0.736
	Median (Min-Max)	29.5 (10.3–49.6)	30.7 (10.8–48.5)	28.7 (10–50)	
**Creatinine (mg/dL)**	Mean ± Sd	0.97 ± 0.18	0.95 ± 0.19	0.95 ± 0.22	0.848
	Median (Min-Max)	1 (0.7–1.3)	0.9 (0.6–1.3)	1 (0.6–1.3)	
**ALT (U/L)**	Mean ± Sd	28.64 ± 10.7	23.67 ± 9.96	24.64 ± 12.24	0.085
	Median (Min-Max)	30.2 (8.6–44.7)	22.7 (8.4–42.4)	22.8 (8.2–44.2)	
**AST (U/L)**	Mean ± Sd	23.03 ± 8.22	24.8 ± 9.15	23.88 ± 8.46	0.629
	Median (Min-Max)	22.7 (10.8–39.9)	27 (10–38.7)	24 (10.4–39)	
**CRP (mg/L)**	Mean ± SD	0.31 ± 0.12	3.10 ± 1.11	5.82 ± 2.09	***0.001*** **
	Median (Min-Max)	0.3 (0.1–0.5)	3.2 (1.1–4.9)	6 (3.1–9.8)	
**Vitamin D** **(ng/mL)**	Mean ± SD	42.7 ± 3.82	20.80 ± 6.34	17.78 ± 5.00	***0.001*** **
	Median (Min-Max)	42.5 (34–51)	20 (6–35)	17 (11–33)	
**PTH (pg/mL)**	Mean ± SD	45.98 ± 8.63	49.09 ± 7.73	51.74 ± 9.86	***0.01*** *
	Median (Min-Max)	48 (25–61)	49.5 (34–65)	55 (31–67)	
**Calcium (mg/dL)**	Mean ± SD	9.28 ± 0.44	9.32 ± 0.23	9.32 ± 0.26	0.827
	Median (Min-Max)	9.3 (8.2–10.2)	9.3 (8.9–10.3)	9.3 (8.6–10)	
**Phosphorus (mg/dL)**	Mean ± SD	3.46 ± 0.21	3.52 ± 0.21	3.51 ± 0.26	0.459
	Median (Min-Max)	3.4 (3–3.9)	3.5 (3.1–4)	3.5 (2.9–4.2)	
**Hemoglobin (g/dL)**	Mean ± SD	13.66 ± 1.7	14.15 ± 1.75	13.78 ± 1.69	0.373
	Median (Min-Max)	13.5 (11.1–16.9)	13.9 (11–16.8)	14 (11–16.9)	
**Hematocrit (%)**	Mean ± SD	40.97 ± 5.09	42.46 ± 5.24	41.34 ± 5.08	0.368
	Median (Min-Max)	40.4 (33.2–50.6)	41.6 (33.1–50.4)	41.9 (33–50.6)	
**WBC (/µL)**	Mean ± SD	7.2 ± 2.13	7.92 ± 2.16	7.43 ± 2.12	0.282
	Median (Min-Max)	6.9 (4.1–11)	7.9 (4.3–11)	7.3 (4–10.9)	
**α-Klotho (pg/mL)**	Mean ± SD	373.71 ± 24.16	294.00 ± 22.2	260.59 ± 24.92	***0.001*** **
	Median (Min-Max)	374.5 (320.9–419.1)	293.7 (250.8–351.1)	260.8 (213.7–324.9)	
**FGF-23 (pg/mL)**	Mean ± SD	55.46 ± 6.73	70.61 ± 7.64	78.56 ± 8.79	***0.001*** **
	Median (Min-Max)	55.8 (40.7–73.9)	71.6 (52.4–84.8)	78 (62.9–96.1)	

One-way ANOVA test and Bonferroni test. * *p* < 0.05; ** *p* < 0.01. SD: standard deviation, Min: minimum, Max: maximum. **AHI**: apnea–hypopnea index, **CRP**: C-reactive protein, **FBG**: fasting blood glucose, **FGF-23**: fibroblast growth factor-23, **HbA1c**: hemoglobin A1c, **HOMA-IR**: homeostasis model assessment for insulin resistance, **OSA**: obstructive sleep apnea, **PTH**: parathyroid hormone, **WBCs**: white blood cells.

**Table 3:** The comparison of FGF-23 and α-Klotho levels with comorbidities.

	**Diabetes Mellitus**	**Mean ± SD**	**Median (Min-Max)**	***p* Value**
**α-Klotho (pg/mL)**	**No (*n* = 71)**	330.55 ± 58.34	344.5 (217.3–419.1)	***0.001*** *
	**Yes (*n* = 62)**	284.46 ± 32.56	280.4 (213.7–373.9)	
**FGF-23 (pg/mL)**	**No (*n* = 71)** **Yes (*n* = 62)**	64.94 ± 13.11 72.13 ± 10.2	62.7 (42.5–96.1) 73.2 (40.7–92.4)	***0.001*** *
	**Obesity**	**Mean ± SD**	**Median (Min-Max)**	***p* Value**
**α-Klotho (pg/mL)**	**No (*n* = 82)**	315.21 ± 53.96	305.4 (213.7–415.5)	0.091
	**Yes (*n* = 51)**	299.19 ± 50.85	285.4 (217.3–419.1)	
**FGF-23 (pg/mL)**	**No (*n* = 82)**	68.29 ± 12.07	69.1 (40.7–93)	0.998
	**Yes (*n* = 51)**	68.29 ± 12.87	69.4 (43.7–96.1)	
	**Hypertension**	**Mean ± SD**	**Median (Min-Max)**	***p* Value**
**α-Klotho (pg/mL)**	**No (*n* = 91)** **Yes (*n* = 42)**	307.56 ± 53.67 312.34 ± 52.57	291.8 (213.7–419.1) 306.3 (224.3–415.2)	0.632
**FGF-23 (pg/mL)**	**No (*n* = 91)** **Yes (*n* = 42)**	69.25 ± 11.79 66.22 ± 13.35	70.3 (40.7–96.1) 67.3 (42.5–93)	0.189
	**Asthma**	**Mean ± SD**	**Median (Min-Max)**	***p* Value**
**α-Klotho (pg/mL)**	**No (*n* = 120)**	309.15 ± 53.69	297.8 (213.7–419.1)	0.958
	**Yes (*n* = 13)**	308.33 ± 50.16	297.8 (241.7–395.4)	
**FGF-23 (pg/mL)**	**No (*n* = 120)**	68.42 ± 12.37	69.2 (40.7–96.1)	0.726
	**Yes (*n* = 13)**	67.15 ± 12.41	69.3 (50–93)	
	**COPD**	**Mean ± SD**	**Median (Min-Max)**	***p* Value**
**α-Klotho (pg/mL)**	**No (*n* = 121)**	311.56 ± 52.35	300.4 (213.7–419.1)	0.086
	**Yes (*n* = 12)**	283.90 ± 57.15	275.5 (223.7–397.6)	
**FGF-23 (pg/mL)**	**No (*n* = 121)**	68.12 ± 12.2	69.3 (40.7–96.1)	0.610
	**Yes (*n* = 12)**	70.03 ± 14.1	68.3 (52.4–91.9)	
	**Thyroid Disease**	**Mean ± SD**	**Median (Min-Max)**	***p* Value**
**α-Klotho (pg/mL)**	**No (*n* = 114)**	307.1 ± 53.69	291.3 (213.7–419.1)	0.298
	**Yes (*n* = 19)**	320.85 ± 49.67	309.7 (219.6–415.2)	
**FGF-23 (pg/mL)**	**No (*n* = 114)**	68.61 ± 12.13	69.3 (40.7–96.1)	0.468
	**Yes (*n* = 19)**	66.38 ± 13.71	66 (43.7–90.9)	

Student’s *t*-test. * *p* < 0.01. SD: standard deviation, Min: minimum, Max: maximum. **FGF-23**: fibroblast growth factor-23.

**Table 4:** The ANCOVA results of FGF-23 and α-Klotho levels.

	β	F	*p*	Model F	Model p	R^2^
**α-Klotho**						
**Age**	0.136	0.524	0.470	54.662	***0.001*** **	0.818
**BMI**	−0.023	0.003	0.960			
**Diabetes Mellitus**	1.587	0.105	0.746			
**Hypertension**	1.421	0.095	0.758			
**Asthma**	−10.860	2.337	0.129			
**COPD**	−18.919	6.677	***0.011*** *			
**Thyroid Disease**	1.573	0.070	0.792			
**Vitamin D**	0.186	0.201	0.655			
**AHI Groups**		45.505	***0.001*** **			
		**Mean ± SD**	**%95 CI**			
*Severe OSA*		262.237 ± 5.320	251.705–272.769			
*Non-severe*		294.792 ± 4.606	285.674–303.911			
*Simple snoring*		371.236 ± 7.784	355.827–386.644			
**FGF-23**						
**Age**	0.119	3.858	0.052	22.581	***0.001*** **	0.649
**BMI**	−0.260	3.220	0.075			
**Diabetes Mellitus**	−2.838	3.260	0.073			
**Hypertension**	−2.556	2.974	0.087			
**Asthma**	1.179	0.266	0.607			
**COPD**	0.811	0.119	0.731			
**Thyroid Disease**	−0.090	0.002	0.962			
**Vitamin D**	−0.178	1.793	0.183			
**AHI Groups**		17.182	***0.001*** **			
		**Mean ± Sd**	**%95 CI**			
*Severe OSA*		77.438 ± 1.711	74.050–80.825			
*Non-severe*		69.788 ± 1.482	66.855–72.721			
*Simple snoring*		57.439 ± 2.504	52.483–62.395			

In the analyses, the simple snoring group was used as the reference category. ANCOVA Test. ** *p* < 0.01; * *p* < 0.05. SD: standard deviation, Min: minimum, Max: maximum. **AHI**: apnea–hypopnea index, **BMI**: body mass index, **COPD**: Chronic Obstructive Pulmonary Disease.

**Table 5:** Correlation analysis.

	α-Klotho (pg/mL)		FGF-23 (pg/mL)	
	r	*p* Value	r	*p* Value
**AHI**	–0.805	***0.001*** *	0.718	***0.001*** *
**ODI**	–0.754	***0.001*** *	0.678	***0.001*** *
ǂ **T90 (%)**	–0.813	***0.001*** *	0.715	***0.001*** *
**Min SpO_2_ (%)**	0.764	***0.001*** *	–0.654	***0.001*** *
ǂ **ESS**	–0.775	***0.001*** *	0.702	***0.001*** *
**Neck circumference (cm)**	0.016	0.845	–0.096	0.242

r: Pearson’s correlation coefficient. ǂ r: Spearman’s correlation coefficient. * *p* < 0.01. **AHI**: apnea–hypopnea index, **ESS**: Epworth Sleepiness Scale, **FGF-23**: fibroblast growth factor-23, **Min SpO_2_**: minimum oxygen saturation, **ODI**: oxygen desaturation index, **T90 (%)**: percentage of sleep time with oxygen saturation below 90%.

**Table 6:** ROC curve analysis of FGF-23 and α-Klotho in severe OSA patients.

	AUC (95% CI)	Cut-Off Point	*p* Value	Sensitivity (%95 CI)	Specificity (%95 CI)	PPV (%95 CI)	NPV (%95 CI)
**α-Klotho (pg/mL)**	0.845 (0.762–0.927)	**≤280.3**	***0.001*** **	84.44 (70.5–93.5)	75 (59.7–86.8)	77.6 (67.1–85.4)	82.5 (70.0–90.5)
**FGF-23 (pg/mL)**	0.735 (0.632–0.838)	**≥75.5**	***0.001*** **	62.2 (46.5–76.2)	72.7 (57.2–85.0)	70.0 (57.8–79.9)	65.3 (55.4–74.1)

DeLong test. ** *p* < 0.01 **CI**: Confidence Interval, **FGF-23**: fibroblast growth factor-23, **PPV**: positive predictive value, **NPV**: negative predictive value.

## Data Availability

The data that support the findings of this study are available on request from the corresponding author. The data are not publicly available, due to privacy or ethical restrictions.
